# The Protective Effect of Cx43 Protein-Mediated Phosphocreatine on Myocardial Ischemia/Reperfusion Injury

**DOI:** 10.1155/2021/8838151

**Published:** 2021-01-22

**Authors:** Chen-xi Wang, Jun-jun Guo, An-jie Di, Yu Zhu, Wei-min Han, An-ran Cheng, Cheng Li, Rui-chan Si, Tian-shu Lan, Ran Zhang, Hong-li Liu, Guo-liang Yan

**Affiliations:** ^1^School of Medicine, Xiamen University, Xiamen 361100, China; ^2^Key Laboratory of Functional and Clinical Translational Medicine, Fujian Province University, Xiamen Medical College, Xiamen 361100, China; ^3^Department of Gynecology, Women and Children's Hospital, School of Medicine, Xiamen University, Xiamen 361100, China

## Abstract

**Objectives:**

To verify the protective effect of phosphocreatine on myocardium in an ischemic model and the possible mechanism of action.

**Methods:**

The model of myocardial ischemia/reperfusion (I/R) was established by the ligation balloon method. 30 SD rats were randomly divided into three groups, *n* = 10 in each group. Sham operation group: the coronary artery was not blocked and observed for 120 minutes. The ischemia/reperfusion (*I*/*R*) group was given ischemia for 30 minutes and ischemia reperfusion for 90 minutes. Phosphocreatine (PCr) group: after 30 minutes of ischemia, the rats were intraperitoneally injected with PCr (200 mg/kg) for 90 minutes. The animal groups of myocardial ischemia/reperfusion model in vitro were the same as those in vivo. The heart was removed by thoracotomy and washed immediately in H-K buffer solution. Then, the heart was installed on the Langendorff instrument. The concentration of PCr perfusion fluid in the PCr group was 10 mmol/L. The changes in coronary blood flow in isolated myocardium were recorded. The heart rate and electrocardiogram were recorded by RM6240BT. At the end of the experiment, myocardial pathological sections and Cx43 immunofluorescence staining were made, and the contents of malondialdehyde (MDA) in myocardial tissue were detected.

**Results:**

Phosphocreatinine treatment improved the myocardial ischemia model, performance in electrocardiogram (ECG) changes (ST segment apparent), and histological changes (decrease in necrotic myocardial cells, inflammatory cell infiltration, and a reduction in myocardial edema). At the same time, MDA decreased, while coronary blood flow and Cx43 expression significantly improved.

**Conclusions:**

Phosphocreatine can improve the electrocardiogram and restore histologic changes in ischemic myocardium and coronary blood flow. The postulated mechanism is by inhibiting the generation of free oxygen radicals and restoring the expression of Cx43 protein.

## 1. Introduction

More people have died because of cardiovascular diseases (CVD) than any other diseases in the world [1]. Ischemic heart disease (IHD) is the most prevalent CVD and is defined clinically as acute myocardial infarction (AMI) or angina pectoris [2]. AMI afflicts human health resulting in millions of deaths worldwide. It is the main factor of chronic heart failure with a mortality rate of approximately 10% [3]. At present, the early opening of the culprit blood vessels is the most effective treatment method for acute myocardial injury. However, the resulting reperfusion injury cannot be averted because of its serious effects [4]. Myocardial ischemia/reperfusion (I/R) injury is a complicated pathophysiological disease process that is associated with various factors and pathways [5–9]. A possible cause of damage induced by I/R is the “reactive oxygen species” (ROS) produced during this process [10]. The overproduction of ROS, namely, hydroxyl radicals, hydrogen peroxide, and superoxide radicals is one of the I/R complications which are able to cause severe damage to reproductive tissues. The elevated levels of ROS result in DNA damage, endothelial destruction, and apoptosis of granulosa cells [11, 12]. Oxidative stress could be originated from the perturbation in the balance of prooxidant and antioxidant agents in which the human body would not be capable of eliminating the excessive amount of ROS from the body [13]. Multiple mechanisms participate in I/R-induced tissue damage, including the increased production of ROS, elevation of proinflammatory mediators, and the initiation of proapoptotic factors in different tissues.

Several studies have reported that testicular ischemia/reperfusion incremented the oxidative stress and decreased the level of “antioxidant enzymes” [10, 14]. The use of tadalafil, verapamil, and the combination of them can protect the testis tissue against IR injury [15].

There are multiple mechanisms that have been proposed to underlie the pathology, including oxidative damage [16, 17], energy metabolic disturbance, and calcium overload [18]. However, the deterioration in intracellular ATP generation is likely to result in repeated ischemia during reperfusion [19]. To lessen the myocardial I/R injury after cardiac arrest, it is important to reduce energy consumption while increasing the energy supply to the myocardium. Prevention and management of myocardial I/R injury is a key step in coronary heart disease surgery and is becoming a major clinical issue in the treatment of IHD.

Exogenous phosphocreatine (PCr), an ATP buffer, is a high-energy phosphate admixture and has been shown to play a protective role in I/R-injured myocardial tissue [20, 21]. However, the mechanism by which it protects ischemia-injured myocardial tissue has not been fully elucidated. The aim of this paper was to explore the protective effects of PCr on myocardial infarction in rats and preliminarily discuss the mechanisms associated with oxidative stress and relevant protein levels.

## 2. Materials and Methods

### 2.1. Animals

60 healthy male SD rats weighing 240–280 g were acquired from Slac Laboratory Animal Co. Ltd. (Shanghai, China). The laboratory animal license number is SCXK (FUJIAN)-2007–0001. The experiment obtained the necessary approval from the Animal Ethics Committee of Xiamen University. With a relative humidity of 55 ± 5% in a pathogen-free facility, the animals were kept under standard laboratory conditions at 22 ± 2°C. All procedures were in accordance with the Institutional Animal Care and Use Committee (IACUC) guidelines and the Laboratory animals guidelines for ethical review of welfare (GB/T 35892–2018).

### 2.2. Drugs and Instruments

Sodium creatine phosphate (PCr), number 922-32-7, was obtained from the Dalian Meilun Biotechnology Co., Ltd. MDA Kits, no. A003-2, were obtained from the Nanjing Jiancheng Bio Co. (Nanjing, China). Antibodies of connexin 43 (Cx43), no. C6219, were obtained from Sigma Bio Co. (MO63103, USA). Krebs-Henseleit fluid (k-h fluid) was composed at a ratio of 120 mmol/L NaCl, 1.2 mmol/L KH_2_PO_4_, 1.2 mmol/L CaCl_2_, 1.2 mmol/L MgSO_4_, 25 mmol/L sodium acetate, and 11 mmol/L glucose, pH 7.4. In the experiment, a mixture of 95% O_2_ and 5% CO_2_ was used. Ringer solution composition was 6.5 g NaCl, 0.12 g CaCl_2_, 0.14 g KCl, 0.10 g Na_2_HPO_4_·12H_2_O, 0.20 g NaHCO_3_, and 2.00 g glucose, in 1.0 L distilled water. Other reagents employed were either domestic or imported analytical reagents.

Multichannel biological signal acquisition instrument (RM6240BT, Chengdu instrument factory, Chengdu, China) and multichannel biological signal acquisition and processing system (version 3.0) were used. A small animal ventilator, number HX-101E, (Chengdu Taimeng Software Co., Ltd., Chengdu, China), was used. A Heart Langendorff irrigation device (number LGF-18, Chengdu instrument factory, Chengdu, China) was used. A visible spectrophotometer (number V-1100D) and MAPADA were also used to assess absorbance.

### 2.3. Model Preparation and Grouping

#### 2.3.1. The Surgical Process of a Myocardial I/R Model

The model of myocardial ischemia reperfusion was established by ligating the left anterior descending (LAD) coronary artery and releasing the ligation line [22, 23]. The blunt head guide wire of the rat's upper teeth was lifted up from the root of the tongue and inserted into the trachea along the direction of cricoid cartilage, and then the ventilator was connected. The rats were anesthetized by intraperitoneal injection of pentobarbital sodium solution (50 mg/kg). The skin between the second and fifth ribs of the left chest was incised with a scalpel, and the subcutaneous tissue, serratus anterior, and pectoralis major muscle were bluntly separated. The chest expander was placed between 2 and 3 ribs to fully expose the heart. The LAD coronary artery was located, the ligation line was 6–0 operation line, the depth of the needle was 2 mm, and the width was 2-3 mm.

#### 2.3.2. Establishment of a Myocardial I/R Model In Vivo

30 SD rats were randomly divided into three groups, *n* = 10 in each group. The control group (sham operation group): the coronary artery was not blocked and observed for 120 minutes. The ischemia/reperfusion (I/R) group was given ischemia for 30 minutes and ischemia reperfusion for 90 minutes. The creatine phosphate (PCr) group: after 30 minutes of ischemia, the rats were intraperitoneally injected with 200 mg/kg PCr (the concentration of PCr was 40 mg/ml) and then given ischemia reperfusion for 90 minutes. The heart rate and electrocardiogram were recorded by RM6240BT. In the experiment, purple cyanosis and electrocardiogram II lead ST elevation in the anterior wall of the left ventricle developed into myocardial ischemia.

#### 2.3.3. Establishment of a Myocardial I/R Model In Vitro

The animal groups of the myocardial I/R model in vitro were the same as those in vivo. The heart was removed by thoracotomy and washed immediately in H-K buffer solution [23]. Then, the heart was installed on Langendorff instrument. The concentration of PCr perfusion fluid in PCr group was 10 mmol/L. The changes in the coronary blood flow (MCF) in isolated myocardium were recorded by collecting cardiac output over 1 min. MCF = cardiac effluent per minute/total ventricular weight. The heart rate and electrocardiogram were recorded by RM6240BT.

### 2.4. Sample Collection and Processing

#### 2.4.1. H&E Immunoflorescence Staining

Specimens from different models were examined. All specimens were implanted in OCT and frozen according to routine histological methods [19]. Slices were stored at −80°C. The specimens from different models were then stained with hematoxylin and eosin (H&E). Frozen tissue slices were removed from −80°C and then incubated at 4°C for approximately 20 minutes. The sections were absorbed using 4% paraformaldehyde for 15 minutes, washed twice in PBS washing solution for 5 minutes, and then rinsed in 0.2% Triton X-100 for 5 minutes. After blocking for 30 minutes with 5% skimmed milk, the sections were then incubated at 4°C overnight in primary antibody: rabbit anti-rat (1 : 10000; Sigma). The sections were then washed three times. With overnight incubation, the sections were incubated in the dark for 1 hour at 37°C with rat anti-rabbit FITC secondary antibody (1 : 300; Invitrogen, Carlsbad, CA). The sections were then sealed with mounting medium encasing and DAPI. The fluorescence microscope was used for observation.

#### 2.4.2. MDA and Detection

For detection of MDA, manufacturer instructions were followed. Briefly, to assess the effect of PCr on oxidative stress, the MDA concentration (nmol/mg) was determined using the thiobarbital acid (TBA) method.

### 2.5. Statistics

All data are presented as the mean ± standard deviation (SD). Electrocardiography parameters were compared using paired *t*-tests, while the remaining data were assessed by one-way analysis of variance (ANOVA). A *P* value < 0.05 was recognized to be statistically significant; *P* < 0.01 and *P* < 0.001 indicated highly significant differences. All analyses were performed using Graph Pad Prism 5® (Graph Pad, Inc., La Jolla, CA, USA) software.

## 3. Results

### 3.1. Electrocardiography Changes

#### 3.1.1. The Electrocardiogram Was Used to Monitor Changes in ECG Caused by Ischemic Reperfusion in Rats

**Figure 1:**
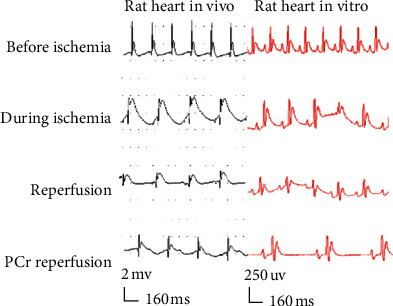
Representative composite electrocardiography changes of rats in vivo and in vitro before ischemia, during ischemia, reperfusion, and PCr reperfusion.

Ischemia immediately induced a progressive increase in ST height and prolongation of the QT segment, and the amplitude of P, *R*, and *T* waves all increased. After ischemia, ST elevation and QT elongation were significantly different from those of the blank control (*P* < 0.001), demonstrating that the myocardial ischemia was obvious after the ligation of the left anterior descending branch. After 10 min of reperfusion, the ST segment height and the QT segment both declined, which proved that the reperfusion was successful. After PCr treatment, the ST segment decreased and the QT segment returned to normal levels. Compared with the control group, there was no statistically significant difference (*P* > 0.05) observed, indicating that PCr exerted a good therapeutic effect on myocardial ischemia. At the same time, in the PCr group, the changes in ST segment, ventricular tachycardia (VT), and ventricular fibrillation (VF) were significantly lower than those of the ischemia/reperfusion group. P, *T*, and *R* waves and the QT interval were significantly smaller than the ischemia/reperfusion group, while the heart rate decreased to a normal level. The results are shown in Figure 1 and Tables 1 and 2.

### 3.2. Pathological Staining of the Heart

**Figure 2:**
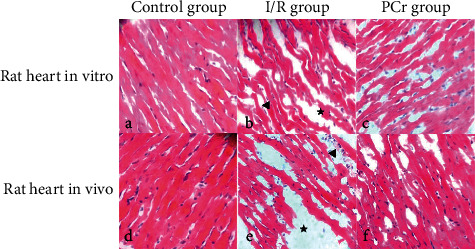
Histopathology of the hearts of rats. Hematoxylin and eosin staining; magnification of 40x ((a–c) rat heart in vitro; (d–f) rat heart in vivo; (a, d), control group; (b, e) I/R group; (c, f) PCr group).

The histologic changes induced immediately after 90 min of reperfusion were evaluated through hematoxylin and eosin staining. Compared to normal myocardial tissue (d, g), myocardial changes were noted immediately after 30 min ischemia. These included coagulation and necrosis, cardiomyocyte disorganization, stromal edema (★), hemorrhage, and increased infiltration of inflammatory cells (◀) (e, h). With the application of PCr, cardiomyocyte necrosis decreased, inflammatory cell infiltration decreased, and there was a relief of myocardial edema (e, i). These data indicated that PCr was able to improve the pathologic damage inflicted upon heart tissue due to myocardial ischemia. The results are shown in Figure 2.

### 3.3. Changes in Coronary Blood Flow in Isolated Rat Hearts

**Figure 3:**
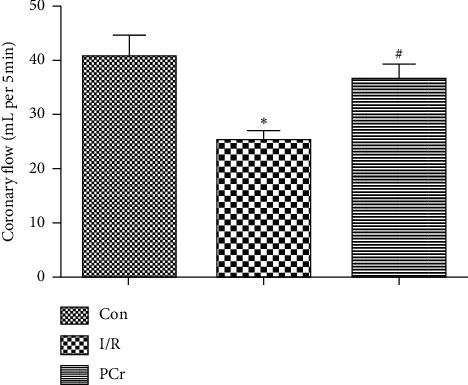
Changes in coronary flow in isolated rat hearts. Compared with the control group, the values in the I/R group had a significantly decreasing trend (*n* = 10, ^∗^*P* < 0.05). Meanwhile, the values in the PCr group had a significantly increasing trend compared with the I/R group (*n* = 10, ^#^*P* < 0.05).

In order to indirectly estimate the contractile capacity of cardiomyocytes after 30 min ischemia, coronary blood flow was assessed once every five minutes using a cylinder to collect blood at the end of the reperfusion period. As shown in Figure 3, the I/R group resulted in a decrease in coronary blood flow compared with the control group, while the PCr group resulted in a significantly increased coronary blood flow compared with the/R group. However, this did not return to the control group level.

### 3.4. MDA Content after Ischemia Reperfusion and PCr Treatment

**Figure 4:**
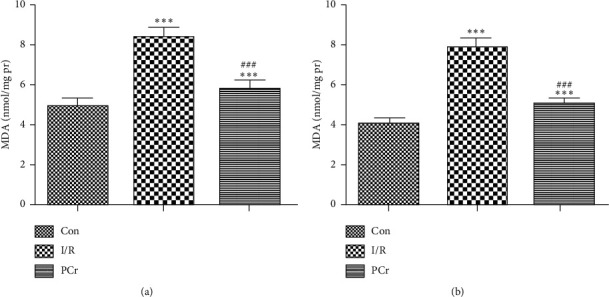
Changes in MDA content. (a) Rat heart in vitro and (b) rat heart in vivo. Compared with the control group, the I/R and PCr groups were significantly different (*n* = 10, ^*∗∗∗*^*P* < 0.001); the PCr group shows a significant difference from the I/R group (*n* = 10, ^###^*P* < 0.001).

As shown in Figure 4, compared with the control group, I/R resulted in higher accumulation of lipid peroxides (*P* < 0.001), while the MDA was significantly decreased compared with the I/R group (*P* < 0.001) but did not return to control levels.

### 3.5. Immunoflorescence Staining of Cx43 in Rat Hearts

**Figure 5:**
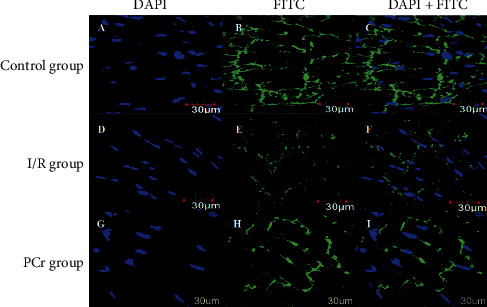
Immunoflorescence staining of Cx43 in rat hearts in vivo (200x). ((a) (b), (c)) Cx43 is mainly located at the intercalated discs of the control group. ((d) (e), (f)) In the I/R group, the Cx43 signal at the intercalated discs is lost and arranged irregularly. ((g) (h), (i)) PCr leads to a partial preservation of Cx43 at the intercalated discs after I/R.

**Figure 6:**
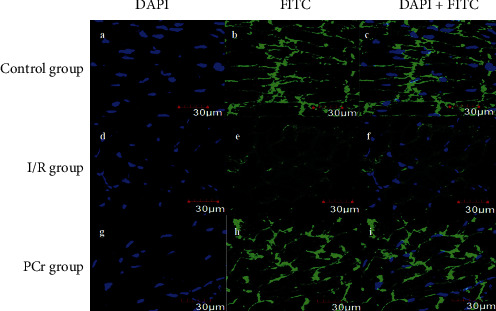
Immunoflorescence staining of Cx43 in rat hearts in vivo (200x). ((a) (b), (c)) Cx43 is mainly located at the intercalated discs of the control group. ((d) (e), (f)) In the I/R group, the Cx43 signal at the intercalated discs is lost and arranged irregularly. ((g) (h), (i)) PCr leads to a partial preservation of Cx43 at the intercalated discs after I/R.

To analyze the distribution of Cx43, heart tissue was labeled with Cx43 antibody and DAPI. In the control group, the nucleus was clearly stained with DAPI, and Cx43 was strongly expressed in the cell membrane (Figures 5(a)–5(c) and Figures 6(a)–6(c)). In contrast, in the I/R group, DAPI staining also showed the nucleus, but the expression of Cx43 in the cell membrane was significantly reduced (Figures 5(d)–5(f) and Figures 6(d)–6(f)). In the PCR group, the recovery of Cx43 expression in the cell membrane was more or less the same as that of the blank control group (Figures 5(g)–5(i) and Figures 6(g)–6(i)). At the same time, the fluorescence intensity was analyzed. Compared with the control group, the fluorescence intensity of the I/*R* group was significantly decreased, and the fluorescence intensity increased after PCr treatment. The results are shown in Table 3.

## 4. Discussion

Ischemic heart disease (IHD) still represents a large burden on individuals and health care resources worldwide. With the recent advances in cardiovascular research, IHD continues to be associated with both high morbidity and mortality globally [24]. IHD pathophysiology is more complex and multifaceted than a single, simplistic, cause-effect event. A large percentage of patients with IHD have minimal or no epicardial coronary vascular disease. Microvascular disease plays an important role in the etiology of IHD, by regulating blood flow and oxygen and energetic substrates delivery, in the microcirculation-myocardium interaction [25]. In the last decades, coronary microcirculation function and structure abnormalities have been described as a relevant IHD pathogenic mechanism. Coronary microvascular dysfunction (CMD) represents a common pathophysiological mechanism of type II myocardial infarction. CMD may have consequences also on hemorheological features of blood flow in epicardial arteries, worsening coronary artery disease (CAD) and establishing a vicious circle, which contributes to myocardial ischemia [26, 27]. CMD determines an inability of the coronary circulation to satisfy myocardial metabolic demands, due to the imbalance of coronary blood flow regulatory mechanisms, including ion channels, leading to the development of hypoxia, fibrosis, and tissue death, which may determine a loss of myocardial function, even beyond the presence of atherosclerotic epicardial plaques. For this reason, ion channels may represent the link among coronary microvascular dysfunction, ischemic heart disease, and consequent heart failure [26]. Myocardial ischemia is directly dependent on an impairment of the cross talk between myocardial energy state and coronary blood flow. The coronary macrovascular and microvascular disease may represent just a portion of the multifaceted pathophysiology of myocardial ischemia [25].

This coupled comorbidity of pathological ischemia and therapeutic reinjury of infarcted myocardium, namely, myocardial ischemia/reperfusion injury (MIRI), is particularly refractory to treatment [28, 29]. Traditionally, MIRI can be due to reactive oxygen and nitrogen species (ROS/RNS) generation, reduced availability of nitric oxide (NO), Ca^2+^ overload, and mitochondrial permeability transition pore (mPTP) opening. From the regulation of the inflammatory-immune response and the energy and metabolism in mitochondria to the epigenetic modification of chromatin, more and more novel molecular targets for MIRI and cardioprotection are being identified [30].

Furthermore, reperfusion therapy promotes the rapid recovery of blood flow to the myocardial ischemic zone but can result in further complications including diminished cardiac contractile and electrical conductivity function, and irreversible tissue necrosis [31]. Therefore, reducing reperfusion injury has become an important target of clinical research.

As a crucial energy substrate, PCr has a dual function of both storing and transporting ATP in energy metabolism. Exogenous PCr adds energy directly to the cell through the PCr/CK system [32]. The pharmacokinetics of PCr displays a biphasic distribution in the blood, where the clearance rate of PCr is initially rapid after injection of a single dose. It is bound strongly to the heart due to its high affinity to the myocardium. Correspondingly, through this direct interaction, exogenous PCr may perform greatly in ischemic myocardium. It has been reported that, during periods of myocardial ischemia in rats, PCr conditioning could advance the levels of intracellular ATP and attenuate metabolic stress [33].

In order to check the protective effect of PCr on the myocardium of ischemic reperfusion injury, models of acute ischemia/reperfusion injury in rats were established to certify ECG and histopathological changes. There is evidence available to demonstrate the efficiency of ECG changes in the diagnosis of acute myocardial ischemia/reperfusion injury [34]. In our experiment, after PCr treatment, the ST segment declined and the QT segment returned to normal levels, demonstrating that PCr exerted a good therapeutic effect on myocardial ischemia. At the same time, in the PCr group, the changes in ST segment, ventricular tachycardia (VT), and ventricular fibrillation (VF) were significantly lower than those of the ischemia/reperfusion group. P, T, and *R* waves and the QT interval were significantly smaller than in the ischemia/reperfusion group, while the heart rate dropped to normal levels.

In addition to I/R, the inflammatory response can exacerbate myocardial injury [35, 36]. Myocardial infarction causes neutrophil infiltration into the ischemic area, and this infiltration can result in myocardial damage [37]. The histological changes to the myocardium after ischemia reperfusion were observed, and myocardial necrosis, tissue edema, and inflammatory cell infiltration were all apparent after myocardial ischemia. With PCr treatment, there were significant morphological differences observed. After PCr treatment, histopathology demonstrated that the myocardial injury could be reversed. Our experimental data are consistent with these phenomena. With the application of PCr, necrotic myocardial cells decreased, inflammatory cell infiltration diminished, and myocardial interstitial edema decreased. These data suggest that PCr could improve pathologic damage to heart tissue, which was precipitated by myocardial ischemia.

Acute ischemia reperfusion myocardial injury may include mechanisms associated with mitochondrial damage and consequent energy metabolic disorders, calcium overload, excitatory amino acid (EAAs) nerve toxicity, the accumulation of oxygen free radicals, and other inflammatory reactions. Among these mechanisms, oxidative stress injury is an important mechanism, which is also a currently researched activity. In the human body, the level of ROS and antioxidant compounds are in equilibrium as the overproduction of ROS results in oxidative stress. Oxidative stress could be originated from the perturbation in the balance of prooxidant and antioxidant agents in which the human body would not be capable of eliminating the excessive amount of ROS from the body [13]. Used to evaluate free radical-mediated myocardial cell injury, MDA is a crucial product of lipid peroxidation [38]. Our study confirmed that PCr inhibits the excessive generation of ROS and plays a protective role in cell damage by inhibiting MDA. In addition, PCr can increase blood flow to the coronary arteries after ischemia/reperfusion injury. These data show that PCr is effective at protecting and aiding the recovery of ischemia reperfusion in the heart.

Recently, the protective mechanism of PCr for myocardial ischemia reperfusion has been extensively researched. As the main connecting protein of cardiomyocytes, Cx43 provides a structural basis for the maintenance of the electrical physiological pulsation of the cardiomyocyte [39, 40]. To explore whether the protective effect of PCr on myocardium after ischemic reperfusion injury was related to the expression of Cx43, we established a myocardial model of rat ischemia/reperfusion injury and immunoflorescence staining to observe changes in Cx43. Our data showed that, in the control group, there was visible staining in the nucleus, Cx43 was expressed on the membrane at higher levels, while, in the I/R group, the expression of Cx43 abundance decreases, which was consistent with overall decreased cardiac function after myocardial ischemia. When the PCr treatment was initiated, the expression of Cx43 protein increased again, and levels approximated that of the control group. This is consistent with the overall recovery of cardiac function after PCr. The aforementioned data imply that the protective mechanism of PCr against cardiac ischemia/reperfusion injury may be directly or indirectly related to the increase and decrease in Cx43 protein expression.

For the first time, this study found that PCr may exert its effect on the myocardium by affecting the expression of Cx43. However, the exact mechanism of action needs further elucidation through future research.

## 5. Conclusion

Our study showed that PCr could be used to treat and improve myocardial ischemia in a model of ischemia/reperfusion injury. The ST segment in the ECG and necrotic myocardial cells decreased, inflammatory cell infiltration decreased, myocardial interstitial edema decreased, and coronary blood flow was restored. One possible mechanism is through inhibition of the generation of oxygen free radicals and to protect the expression of Cx43 protein, thus restoring normal function to the myocardium.

## Figures and Tables

**Table 1:** Electrocardiographic parameters as calculated from the composite recordings in rats in vivo (*n* = 10).

Group	Heart rate (BMP)	ST height (mV)	QT (ms)	P amplitude (mV)	R amplitude (mV)	T amplitude (mV)
Control	356.1 ± 40.2	14.5 ± 10.7	76.7 ± 14.1	2.77 ± 1.53	6.04 ± 1.55	2.03 ± 1.02
I/R	236.5 ± 42.1^*∗∗*^	200.7 ± 33.2^*∗∗∗*^	105.4 ± 17.3^*∗*^	5.84 ± 2.58^*∗*^	8.82 ± 2.01^*∗*^	3.72 ± 1.17^*∗*^
PCr	313.1 ± 37.2^#^	15.3 ± 12.6^###^	80.2 ± 14.3^#^	3.04 ± 1.65^#^	6.22 ± 1.23^#^	2.08 ± 1.04^#^

Note: ^*∗*^*P* < 0.05,^*∗∗*^ < 0.01,^*∗∗∗*^ < 0.001 versus the control group. ^#^*P* < 0.05, ^##^*P* < 0.01, ^###^*P* < 0.001 versus the I/R group. PCr group measurements were not all statistically significant, compared with the control group (*P* > 0.05).

**Table 2:** Electrocardiographic parameters as calculated from the composite recordings in rats in vitro (*n* = 10).

Group	Heart rate (BMP)	ST height (mV)	QT (ms)	P amplitude (mV)	R amplitude (mV)	T amplitude (mV)
Control	267.3 ± 32.4	20.5 ± 10.4	82.1 ± 21.3	2.61 ± 1.37	6.11 ± 1.74	1.98 ± 1.09
I/R	166.6 ± 30.8^*∗∗*^	280.7 ± 43.5^*∗∗∗*^	144.0 ± 35.4^*∗*^	5.77 ± 2.49^*∗*^	8.95 ± 2.17^*∗*^	3.87 ± 1.23^*∗*^
PCr	254.7 ± 25.2^##^	25.3 ± 22.6^###^	97.3 ± 19.8^#^	2.95 ± 1.54^#^	6.25 ± 1.35^#^	2.18 ± 1.03^#^

Note: ^*∗*^*P* < 0.05,^*∗∗*^*P* < 0.01,^*∗∗∗*^ < 0.001 versus control group. ^#^*P* < 0.05, ^##^*P* < 0.01, ^###^*P* < 0.001 versus I/R group. PCr group measurements were not all statistically significant, compared with the control group (*P* > 0.05).

**Table 3:** The results of fluorescence intensity analysis.

No.	*B*	*E*	*H*	*b*	*e*	*h*
Constituency area	1.85	1.63	1.85	1.94	1.94	1.94
Average fluorescence intensity	14.28	4.46	8.63	14.85	4.16	13.88

## Data Availability

The data used to support the findings of this study are available from the corresponding author upon request.
